# The Test Based on Meta-Analysis on “Does Workaholism Prefer Task Performance or Contextual Performance?”

**DOI:** 10.3389/fpsyg.2022.860687

**Published:** 2022-05-02

**Authors:** Bang Cheng, Jiajun Gu

**Affiliations:** ^1^School of Economics and Management, Zhejiang Post and Telecommunication College, Shaoxing, China; ^2^School of Management, Zhejiang Gongshang University Hangzhou College of Commerce, Hangzhou, China

**Keywords:** workaholism, working excessively, working compulsively, work performance, meta-analysis

## Abstract

The relationship between workaholism and work performance is explored by meta-analysis in this article. After searching relevant references, we had gained 94 individual effect sizes (*n* = 57,352), 45 individual samples, and 37 references. Through the heterogeneity test, it was shown that the random effect model is more suitable. The main effect analysis showed that there is a significant positive correlation between workaholism, working excessively, working compulsively, and work performance, and further analysis showed that workaholism emphasizes the improvement of contextual performance. The subgroup test showed that the relationship between workaholism, working excessively, working compulsively, and work performance is influenced by the measurement tools of workaholism, but not influenced by the cultural background differences and time-lag research. The above results show that workaholism and its dimensions have different influences on different aspects of work performance. Besides, it is worthy to consider the moderating function of the measurement tools of workaholism in the relationship between workaholism and work performance.

## Introduction

With intensified competition in the world and the rapid iteration of Internet technology, the political and economic environment requires employees to devote more time and energy to their work, resulting in a more widespread phenomenon of workaholism. The data show that in 2016 there were 488 million people who worked more than 55 h per week, with a proportion of 8.9%. The proportion in Southeast Asia reached 11.7% (Pega et al., 2021), as it increased rapidly in recent years (especially the home office caused by COVID-19). Researchers treat workaholism as addictive behavior, for instance, alcohol addiction. Data shows that around 7.3–8.3% of Norwegians are addicted to work (Andreassen et al., 2012). The phenomenon of workaholism in Hungary, France, and South Korea reached 20.6 (Orosz et al., 2016), 20.8, and 39.7%, respectively (Kang, 2020). In the United States, one in ten adults falls into the phenomenon of workaholism (Sussman, 2012). From this, we can see that, with the development of society, there has been an increase in workaholism (Ng et al., 2007; Clark et al., 2016). Scholars have also studied widely on this phenomenon.

Generally, we understand workaholism as a phenomenon of the employees’ overwork due to their inner compulsion (Schaufeli et al., 2008), which has been widely recognized by current scholars. However, the scholars have not reached a consensus on whether workaholism is a positive or negative phenomenon (Ng et al., 2007). The scholars have focused on the issue of whether workaholism is synonymous with a high performance of the employees (Machlowitz, 1980; Bonebright, 2001) in the early stage of research, and conducted a great deal of research on the relationship between workaholism and work performance. However, only inconsistent or even opposite conclusions have been reached (Clark et al., 2016), and grasping the essence of the issue will play a positive and important role in correctly treating the phenomenon of workaholism. Some scholars proposed that workaholism has a positive correlation with work performance due to long working hours and high human capital investment (Peiperl and Jones, 2001; Krulder, 2010; Serrano, 2015). There are some studies showing that workaholism and inner compulsion will lead to excessive loss of individual resources, which is unfavorable to the work performance (Shimazu et al., 2015; Aziz et al., 2020). Therefore, what is the true relationship between workaholism and work performance? And is this effect consistent for different types of performance (e.g., task performance and contextual performance)? This is still an issue demanding prompt solution.

To solve the above controversies, we would like to draw more generally recognized and accurate conclusions from a macro-perspective, and deeply explore the relationship between workaholism and work performance based on meta-analysis. Although some foreign scholars have explored the relationship between workaholism and work performance based on meta-analysis (Clark et al., 2016), they only included the relevant literature before 2013 (*k* = 12) with few samples (*n* = 6,726). Numerous researches on the relationship between workaholism and work performance have come out recently (Shimazu et al., 2015; Sandrin et al., 2019; Balducci et al., 2020). It will be helpful to further explore the relationship between them with more samples. Besides, the article in the meta analysis of Clark et al. (2016) only included the population samples of the western countries, without the sample data of the eastern countries, including China, Japan, and South Korea. Therefore, whether the research conclusion is universal remains to be investigated. Finally, the emphasis of scholar of Clark et al. (2016) was not on exploring the above-mentioned relationship. They preliminarily analyzed the overall relationship between workaholism and work performance, and did not comprehensively study the relationship between the subdimensions of workaholism and the different types of work performance and whether they would be influenced by the potential moderator variables. The related studies have shown that there are different relationships between different dimensions of workaholism and types of work performance in the past few years.

For all the above reasons, the article intends to adopt the meta-analysis methods to explore the relationship between workaholism and work performance and the potential moderator variables of the relationship between them. Through the integration of relevant empirical research results in the past, we would like to ensure that the influence of measurement errors caused by single research will be avoided (Wilson and Lipsey, 2001), which is helpful to get a more clear, specific, and universal research conclusion on the relationship between workaholism and work performance.

### Concept and Measurement

Oates (1971) was the first one to propose the concept of workaholism, which means that the work demand of individuals has been overbalanced and has caused an obvious interference on their physical health, personal happiness, interpersonal relationships, and social functions. The followed scholars mainly studied the concept and measurement from two different views: the addiction model and theory of motivation-cognition-behavior-emotion. Seeing from the addiction model, workaholism is deemed as an addictive behavior, featured with six typical characteristics of addiction, including salience, tolerance, mood modification, relapse, withdrawal symptoms, and conflict (Griffiths, 2005). The representative measurement tools mainly involve the work addiction risk test (WART) scale of Robinson (1999), including the compulsive tendencies, control, impaired communication/self-absorption, and inability to delegate and self-worth. Some scholars prefer to take the whole as a dimension (Kun et al., 2020) or choose the compulsive tendencies as the core content (Lee and Choi, 2015; Lanaj et al., 2021). There are 7 items in the Bergen Work Addiction Scale (BWAS) of Andreassen et al. (2012). However, many scholars regard workaholism as a multidimensional concept composed of one or more factors, including motivation, behavior, cognition, and emotion, which will be reflected in the following representative views. Spence and Robbins (1992) divided workaholism into three dimensions, which include job involvement (behavioral), driven (cognitive), and enjoyment of work (emotional), and they proposed that workaholism should achieve high scores in behavior and cognition and low scores in emotion. Schaufeli et al. (2008) divided workaholism into two dimensions, which include working excessively and working compulsively, and constructed the Dutch Workaholism Scale (DUWAS) (Schaufeli et al., 2009b). Aziz et al. (2013) incorporated the concepts of driven and work-family conflicts and prepared a Workaholism Analysis Questionnaire (WAQ), which can effectively recognize the workaholism of the employees. Clark et al. (2020) summarized the above concepts and incorporated the motivation to develop a Multidimensional Workaholism Scale (MWS) for the past few years. They believed that workaholism was a multidimensional structure, including (1) the inner compulsion to work (motivation); (2) thoughts about work (cognitive); (3) negative emotions when not working (emotional); and (4) excessive work behaviors (behavioral). There is no agreed definition on the concept and dimension division of workaholism, but the key dimensions of working excessively and working compulsively are generally recognized (Ng et al., 2007; Clark et al., 2016; Acosta-Prado et al., 2021). The article mainly adopts the dimension division of Schaufeli et al. (2008) to comprehensively explore the relationship between working excessively (behavioral) and working compulsively (cognitive) and work performance, influenced by a few types of researches on the motivation of workaholism and the general recognition of the positive relationship between the emotion dimension and work performance.

Work performance refers to the unity of work behavior and work results of employees in an organization (Borman and Motowidlo, 1993). Borman and Motowidlo (1993) divided the work performance into task performance and contextual performance. Task performance refers to the behaviors of providing products or services directly related to work. Contextual performance refers to the behaviors and efforts that are not directly related to their work, including organizational citizenship behavior and prosocial behavior. The model has also been widely recognized and used by scholars, which is also studied by the article by its concept and theoretical model.

### Relationship Between Workaholism and Work Performance

Scholars have carried out numerous researches on the relationship between workaholism and work performance, but there is no consensus on its conclusion. Currently, there are mainly three views from research, including a positive view, negative view, and insignificant view. Scholars with a positive view regard the workaholics as hyper-performers with high productivity, who seek enthusiasm and psychological satisfaction by participating in work through highly efficient work and long-time energy devotion, to obtain high-performance reports (Machlowitz, 1980; Peiperl and Jones, 2001; Ng et al., 2007; Golzari et al., 2012; Serrano, 2015). Shimazu and Schaufeli (2009) found that the direct effect of workaholism and work performance was not significant, but its positive effect of indirectly influencing work performance through positive coping has been confirmed. Scholars with negative views think that workaholics work hard but are not smart enough, are perfectionists, rigid, inflexible with work arrangements, and reluctant to share with others. Therefore, their performance will not be too high (Oates, 1971; Van Beek et al., 2014; Birkeland and Buch, 2015; Aziz et al., 2020). From the views of the conservation of resources theory (COR) and the effort-recovery model, some scholars believe that workaholics without enough time to rest and recover due to the loss of numerous cognitive and emotional resources (Falco et al., 2013), an increase of physical and mental stress, and perceivable increase of work demand (Alessandri et al., 2020), show the appearance of negative emotions (Gorgievski et al., 2014), which lead to emotional exhaustion (Sandrin et al., 2019; Balducci et al., 2020). The employees under such status tend to protect resources, and are not willing and do not have enough resources to carry out internal or additional work (Aziz et al., 2020). Some scholars also proposed that the relationship between workaholism and work performance is not significant (Birkeland and Buch, 2015; Shimazu et al., 2015; Clark et al., 2016; Sandrin et al., 2019; Balducci et al., 2020). The reason may be that working compulsively offsets the positive effects of working excessively and the enjoyment of work (Gorgievski et al., 2010). Therefore, the performance of workaholics has not improved significantly (Van Beek et al., 2013; Shimazu et al., 2015; Spagnoli et al., 2020). These factors play a vital role in the work performance of the workaholics and most researchers believe that working excessively can significantly improve work performance while working compulsively will function in the opposite way (Shimazu and Schaufeli, 2009).

In the followed study, the scholars found that the relationship between workaholism and work performance is also different. Schaufeli et al. (2006) found that workaholism (working excessively and working compulsively) will improve the external role behavior, namely the contextual performance (Van Beek et al., 2014), which also indirectly confirms that the workaholics exceed the reasonable requirements of work or organization. Furthermore, some scholars proposed that the reason workaholics have a higher contextual performance is because workaholics are more willing to contribute and show more innovation behaviors (Gorgievski et al., 2010, 2014). The other scholars proposed that the relationship between workaholism and work performance is influenced by other factors, including the enjoyment of work, work engagement, supervisor appraisal, job satisfaction, and types of workaholics (Bonebright, 2001; Graves et al., 2012; Mazzetti et al., 2016; Spagnoli et al., 2020). Spagnoli et al. (2020) proposed that when the work engagement of the workaholics is at a low level, the negative relationship with work performance will be highlighted. As for the relationship between workaholism and work performance, this research shows that it mainly depends on the influence of its subdivision of working excessively and working compulsively on work performance.

Therefore, the following assumptions are proposed in the article according to the above discussion:

H1: There is a positive correlation between working excessively and work performance, task performance and contextual performance.

H2: There is a negative correlation between working compulsively and work performance, task performance and contextual performance.

H3: There is no significant relationship between workaholism and work performance.

### Moderator Variables

By analyzing the references, it is found that there are inconsistent conclusions on the relationship between workaholism and work performance. Therefore, we have reasons to believe that there are potential moderator variables between workaholism and work performance. After more detailed and comprehensive research on the relevant references, the meta-analysis regards the measurement tools of workaholism, cultural background, and time-lag research as moderator variables.

According to the studies in the past, different researchers held different definitions and dimensions for workaholism so they adopted different measurement tools, mainly reflecting the inconsistencies in the tools and dimensions. First, the measurement tools are inconsistent. There are many different measurement tools, including the WorkBAT scale of Spence and Robbins (1992), the WART scale of Robinson (1999), the DUWAS scale developed by Schaufeli et al. (2009a), the WAQ questionnaire of Aziz et al. (2013), and the BWAS scale developed by Andreassen et al. (2012). However, there are various adapted versions suit able local culture of the same scale. For example, the WorkBAT-R and JVCT scales come from the WorkBAT scale. Therefore, there are great differences in the measurement tools so it is difficult to keep the same measurement content (Andreassen et al., 2013; Acosta-Prado et al., 2021). Second, the dimension divisions are inconsistent. The scholars hold different dimensions of workaholism based on different research focuses and workaholism models. For instance, Spence and Robbins (1992) analyzed workaholism from three dimensions: involvement, driven, and enjoyment of work. The WorkBAT–Rand JVCT scales excluded job involvement and only kept driven and enjoyment of work scales. In contrast, Schaufeli et al. (2009b) regarded workaholism as a combination of working excessively and working compulsively, and excluded enjoyment of work. Robinson (1999) and Andreassen et al. (2012) developed the workaholism scale from the perspective of addiction. The difference is that there are five dimensions in the WART scale of Robinson (1999), while there is only one dimension in the later BWAS scale. The inconsistency of the measurement tools will directly influence the strength and direction of the relationships among the variables. Therefore, this research assumes that the relationship between workaholism and work performance may be moderated by different measurement tools of workaholism. In conclusion, we propose the following assumptions:

H4: The measurement tools of workaholism can moderate the relationship between workaholism and work performance.

In terms of the cultural background, we divide cultural values into individualism and collectivism viewed at the national level according to the divisions by Hofstede et al. (2010). According to the coding method of Yang and Li (2021), the countries with an individualism index beyond 50 are deemed as countries with an individualism cultural tendency, highlighted by the United States, France, Germany, and Spain. The countries with the individualism scores below 50 are deemed as countries with collectivism cultural tendencies, highlighted by China, Japan, South Korea, Iran, and Turkey. Next, we would like to briefly describe the influence of cultural background on the relationship between workaholism and work performance. First, workaholism is closely related to cultural background (Hu et al., 2014). Under the tendency of individualism culture, personal self-realization is highly appreciated, and the workaholics are mainly driven by internal motivation, representing a low degree of job involvement and drive, and a high degree of enjoyment of work. On the contrary, the social value system filled with diligence, effort, and responsibility is highly appreciated under collectivism with high popularity of workaholism. Studies in the past have also shown that cultural background influences the degree of workaholism (Shkoler et al., 2017). Generally, workaholism in eastern countries is more popular than that in western countries (Hu et al., 2014). Compared with the individualism culture, employees care more about their long-term development in the high collectivism environment. In return, they care about their work performance (including the improvement of task performance and contextual performance) (Jackson et al., 2006). Finally, nationality or cultural background also plays a role in the relationship between workaholism and the psychological and behavioral results of the employees. Therefore, we propose the following assumptions:

H5: Cultural background can moderate the relationship between workaholism and work performance. Workaholism under the individualism culture shows a higher connection with work performance.

The time-lag research is mainly carried out from cross-sectional research and longitudinal research. Some studies have shown that longitudinal research can better reflect the causal relationship between variables. Ng et al. (2007) proposed that the relationship between workaholics and work performance may be influenced by time. Seeing from short-term, work performance will be improved by the numerous devotions with the cost of workaholics. However, seeing from long-term, work performance will be damaged due to the increase of psychological and physical costs and the emergence of health and cognitive–emotional problems. Relevant studies also indirectly proved the view. Gorgievski et al. (2010) found that work performance can be significantly improved by the workaholics in short-term research. Through a 2-year longitudinal study, Shimazu et al. (2015) found that workaholism will not lead to the improvement of work performance. Therefore, we propose the following assumptions:

H6: The time-lag research plays a moderating role between workaholism and work performance. The data show that there is a stronger relationship if studied longitudinally.

## Research Methods

### References Search

**FIGURE 1:**
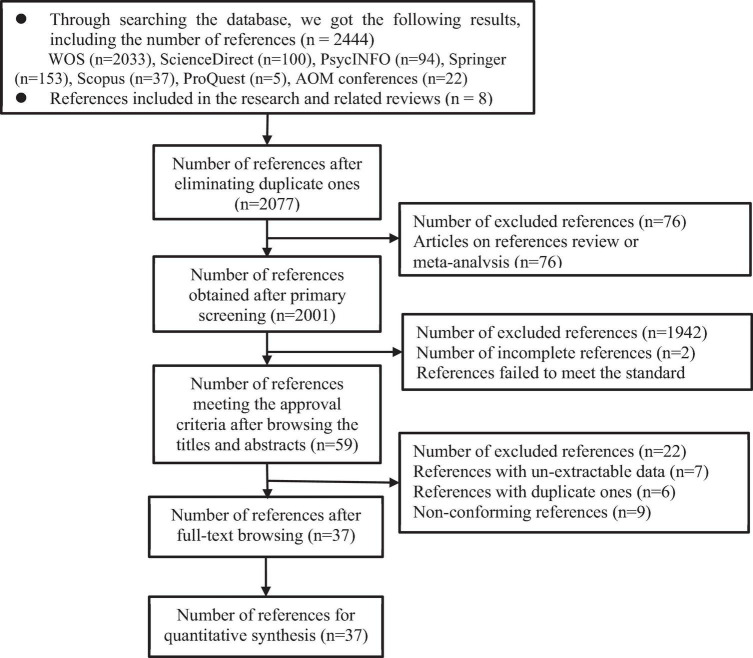
Flow chart of the searching and screening of references.

The article mainly searched the English database, including Web of Science, Elsevier ScienceDirect, ProQuest Database, PsycINFO, Springer Link, Scopus, and Wiley. The search words of workaholism are workaholism, workaholic, and work addiction. The keywords of work performance are performance, organizational citizenship behavior, productivity, and innovativeness. The keywords of academy of management (AOM) and society for industrial and organizational psychology (SIOP) related to conferences are searched in the article. Finally, the article conducted a supplement searching with the references in Google Scholar. For searching more comprehensive relevant references, the article also reviewed the references searched through the above ways, and analyzed the references on workaholism (Ng et al., 2007; Sussman, 2012; Clark et al., 2016), to supplement for the possible insufficient references. The searching and screening process for specific references is shown in Figure 1.

### Inclusion and Exclusion Criteria of References

Combining requirements of the meta-analysis technics and related research topics, the meta-analysis includes the following criteria: (1) They must be empirical research articles; references reviews and theoretical articles are excluded. (2) The correlation coefficient between the dimension of workaholism and work performance of the same individual or the indicators that can transform the effect size (value of t and F, ΔR^2^). (3) The samples of each research shall be independent of each other. If the samples are repeated, the studies with larger sample size or more detailed sample information shall be included. (4) If the research is published repeatedly, one of them will be valid. If the dissertation is published in journals, academic journals are preferred. (5) The object of the research must be the staff of the enterprises or institutions, so the student group is excluded. (6) the sample size must be reported.

### Document Coding

The references included in the meta-analysis study shall be coded with the following information: basic information of the references (author + publication time), dimensions of workaholism (workaholism, working excessively, and working compulsively), measurement work of workaholism (WorkBAT, DUWAS, WART, others), cultural background (individualism, collectivism), time-lag study (cross-sectional study, longitudinal study). The effect size of one reference corresponds to an independent research sample. If multiple independent samples are reported in the same research, they shall be coded separately to generate multiple independent effect sizes, with a total of 94 independent effect sizes. According to the inclusion criteria of the references, the second author and the third author shall code independently, and reach the 95.7% coding consistency. The coding differences shall be discussed and integrated by the first author, which shows that the coding process is relatively effective and accurate. A total of 37 references were finally included in the analysis, as shown in Table 1.

**TABLE 1:** Basic data of meta-analysis research.

Author (released time)	Quantities	Tools	Culture	Time-lag study	Relationship of variables	Effect size (r)
Aksoy and Yalçınsoy (2018)	189	WorkBAT	Co	Cross-section	WA&WP	−0.037
Alessandri et al. (2020)	510	DUWAS	In	Longitudinal	WA&CP	−0.21
Alonderienė et al. (2017)	297	WorkBAT	In	Cross-section	WE&TP, WC&TP	0.093, 0.114
Aziz et al. (2020)	409	WAQ	In	Cross-section	WA&CP	−0.25
Balducci et al. (2020)	519	DUWAS	In	Longitudinal	WA&TP	0.01, 0.12
Birkeland and Buch (2015)	175	DUWAS	In	Longitudinal	WA&TP, WA&CP	0.10, −0.04
Bonebright (2001) E1	123	WorkBAT	In	Cross-section	WE&TP, WC&TP	0.02, −0.11
Bonebright (2001) E2	45	WorkBAT	In	Cross-section	WE&TP, WC&TP	−0.13, −0.03
Bonebright (2001) E3	71	WorkBAT	In	Cross-section	WE&TP, WC&TP	0.27, 0.03
Bonebright (2001) E4	78	WorkBAT	In	Cross-section	WE&TP,WC&TP	0.16, 0.11
Clercq (2017)	120	DUWAS	In	Cross-section	WA&WP, TP, CP	0.314, 0.37, 0.176
Falco et al. (2013)	322	DUWAS	In	Longitudinal	WA&TP	−0.02
Gillet et al. (2021)	419	DUWAS	In	Cross-section	WA&TP	−0.054
Golzari et al. (2012)	200	WorkBAT	Co	Cross-section	WE&CP, WC&CP	0.64, 0.47
Gorgievski et al. (2010) E1	262	DUWAS	In	Cross-section	WE&TP, WE&CP, WE&CP, WC&TP, WC&CP, WC&CP	0.22, 0.28, 0.32, 0.04, 0.03, 0.02
Gorgievski et al. (2010) E2	1,900	DUWAS	In	Cross-section	WE&TP, WE&CP, WE&CP, WC&TP, WC&CP, WC&CP	0.11, 0.31, 0.40, 0.02, 0.22, 0.16
Gorgievski et al. (2014)	180	DUWAS	In	Cross-section	WA&TP, WA&TP, WA&CP	−0.11, −0.09, 0.25
Graves et al. (2012)	357	WorkBAT	In	Cross-section	WC&TP, WC&TP, WC&CP	−0.02, 0.05, −0.04
Hung (2018)	300	DUWAS	Co	Cross-section	WA&TP, WA&CP	0.567, 0.55
Jackson (2011)	530	WorkBAT	In	Cross-section	WA&WP, WA&WP	0.363, 0.21
Krulder (2010)	1,325	DUWAS	Co	Cross-section	WE&TP, WE&CP, WC&TP, WC&CP	0.12, 0.18, 0.11, 0.10
Lanaj et al. (2021)	645	WART	In	Longitudinal	WC&TP, WC&CP	0.001, 0.12
Laurence (2011)	163	WorkBAT	Co	Cross-section	WC&TP, WC&CP	0.156, −0.121
Lee and Choi (2015)	178	WART	Co	Cross-section	WE&CP, WC&CP	−0.31, 0.34
Mazzetti et al. (2016)	295	DUWAS	In	Cross-section	WA&TP	0.04
Nastasi (2018)	1,000	BWAS	In	Cross-section	WA&WP	−0.04
Robledo et al. (2019)	443	DUWAS	In	Longitudinal	WA&TP, WE&TP, WC&TP	−0.13, −0.12, −0.12
Sandrin et al. (2019)	1,028	DUWAS	In	Cross-sectional	WA&TP	−0.14
Schaufeli et al. (2009a)	2,115	DUWAS	In	Cross-sectional	WE&TP, WC&TP	−0.28, −0.22
Serrano (2015) E1	214	DUWAS	In	Cross-sectional	WA&TP	0.25
Serrano (2015) E2	255	DUWAS	In	Cross-sectional	WA&TP	0.32, 0.47
Serrano (2015) E3	255	DUWAS	In	Longitudinal	WA&TP	0.4
Shimazu and Schaufeli (2009)	776	DUWAS	Co	Cross-sectional	WE&TP, WC&TP	0.01, −0.08
Shimazu et al. (2012)	1,967	DUWAS	Co	Longitudinal	WE&TP, WE&CP, WC&TP, WC&CP	0.04, 0.06, 0.02, 0.04
Shimazu et al. (2015)	1,196	DUWAS	Co	Longitudinal	WE&TP, WE&CP, WC&TP, WC&CP	0.05, 0.11, 0.08, 0.08
Smith et al. (2020) E1	292	WART	In	Cross-sectional	WC&TP, CP, CP	0.13, 0.18, 0.32
Smith et al. (2020) E2	162	WART	In	Longitudinal	WC&CP, CP	0.18, 0.31
Sok et al. (2018)	534	WorkBAT	In	Cross-sectional	WC&TP	0.01
Spagnoli et al. (2020)	208	BWAS	In	Longitudinal	WA&TP	−0.15
Stock and Bauer (2011)	224	WorkBAT	In	Cross-sectional	WC&WP	−0.23
Van Beek et al. (2014) Y1	680	DUWAS	In	Cross-sectional	WE&TP, WC&TP	−0.07, −0.10
Van Beek et al. (2014) Y2	275	DUWAS	In	Cross-sectional	WE&TP, WE&CP, WC&TP, WC&CP	−0.15, 0.21, −0.1, 0.13
Xu et al. (2021)	700	DUWAS	Co	Cross-sectional	WA&TP	0.17
Zhang et al. (2021)	254	DUWAS	Co	Cross-sectional	WA&TP	0.5

*(1) In = individualism cultural tendency, Co, collectivism cultural tendency; (2) WA, workaholism; WE, working excessively; WC, working compulsively; TP, task performance; CP, contextual performance; (3) If there are different samples included in the same research, which are distinguished by E1, E2, E3; (4) If the first author is the same and the year is the same, they will be distinguished by Y1, Y2.*

### Meta-Analysis Process

#### Meta-Analysis Program

First, the article makes the calculation by the CMA (Comprehensive Meta-analysis 3.0) software, mainly including the effect value conversion, publication bias test, heterogeneity test, main effect and moderator effect test. Before calculation, the correlation coefficient is corrected first according to the correction formula ES_r_* = ES_r_/$\sqrt{r_{x\,x}\,r_{y\,y}}$, aiming at avoiding the deviation of correlation-coefficient-caused insufficient reliability, where ES_r_ represents the correlation coefficient of the variables, r_xx_ and r_yy_ represent the reliability of the corresponding variables, and the reliability coefficient is expressed by 1 (Hunter and Schmidt, 2004) for the variable of a single item. After the above coefficient conversion, subsequent analysis is conducted on the CMA software.

#### Model Selection and Heterogeneity Test

There are two meta-analysis models now, including the fixed effect model and random effect model. The fixed effect model assumes that the results of the same study are true, and the difference is caused by a sampling error. There is an effect quantity in all studies, but this effect quantity cannot be extended to the other populations. While, the random model believes that there are different effect quantities in different studies, which may be caused by different studies’ methods and sample populations (Borenstein et al., 2009). When analyzing the relevant references on workaholism and work performance, the author found that the relationship between them may be influenced by the measurement tools of workaholism, cultural background, and time-lag research. The random effect model (Borenstein et al., 2009) shall be selected, if the meta-analysis results are influenced by different research characteristics. This is the reason the article adopts the random effect model for meta-analysis. In addition, the article further verifies the rationality of selecting the fixed effect model through the heterogeneity test. The heterogeneity test methods mainly include the forest plot, *Q*-test, *I*^2^-test, and *H*-test. The forest map is a subjective judgment based on graphics. The *Q*-test is the comparison of the standardized weighted total variance and expected total variance, *p* < 0.1 (0.05) indicates the existence of heterogeneity. The *I*^2^-test is the ratio of inter-study variance to total variance; 25, 50, and 75% can be deemed as the boundary of a low, medium, and high heterogeneity (Higgins et al., 2003). The *H*-test is the correction value of the *Q*-test. If H > 1.5, it indicates that there is a high heterogeneity between the studies.

#### Publication Bias

Publication bias means that the published studies cannot completely replace all completed studies (Rothstein et al., 2005). The unpublished articles with insignificant results will influence the reliability of the meta-analysis results, resulting in the over evaluation of the original effect, and the effective measure to avoid publication bias is to increase the sample size. The article turned to scholars in relevant fields for unpublished data managing to collect more comprehensive literature as much as possible during references search to avoid this problem, as shown in Table 1. At the same time, the funnel plot, Rosenthal’s Classic Fail-safe *N* test, and Egger’s test were used to further test the publication bias in the followed analysis.

## Research Results

### Publication Bias Test

**FIGURE 2:**
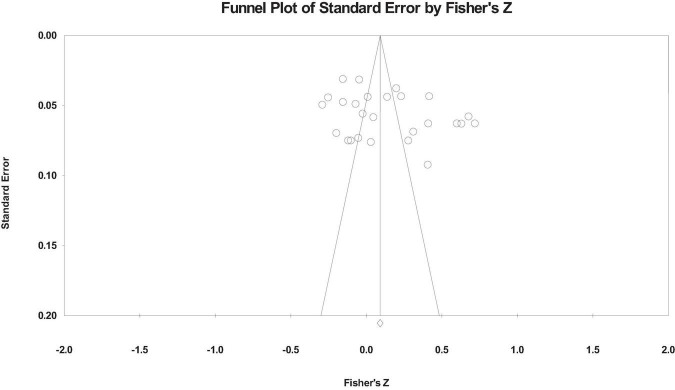
Funnel plot of workaholism on the work performance.

**FIGURE 3:**
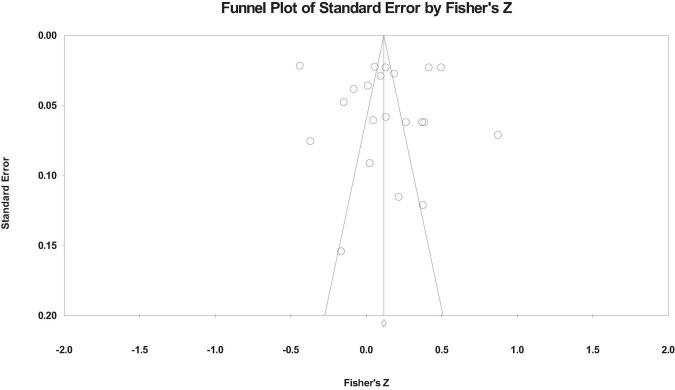
Funnel plot of working excessively on the work performance.

**FIGURE 4:**
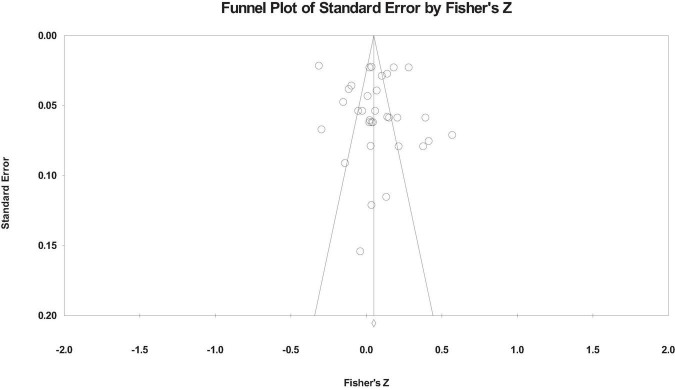
Funnel plot of working compulsively on the work performance.

The funnel plot is used to test the publication bias, as shown in Figures 2–4. From the funnel plot, we can see that the related references on the relationship between workaholism and its dimensions and work performance is distributed on both sides of the total effect size, and the results show that there is no serious publication bias (Borenstein et al., 2009). However, the funnel plot is a subjective method to test publication bias. Therefore, we also used Rosenthal’s Classic Fail-safe N, Begg and Mazumdar rank correlation, and Egger’s test to test the publication bias. Table 2 shows the publication bias test results.

**TABLE 2:** Test results of publication bias.

Variables relationship	K	Classic fail-safe N	Value *P*	Begg and Mazumdar rank correlation		Egger’s value *P*
				Uncorrected Value *P*	Corrected Value *P*	
WA → WP	26	845	0.000	0.300	0.311	0.091
WE → WP	21	1,055	0.000	0.856	0.880	0.797
WC → WP	33	485	0.000	0.566	0.577	0.479

*K is the number of independent samples involved. The same below.*

The results show that the fail-safe coefficients of the total scores of the funnel plot, workaholism, working excessively, and working compulsively on the work performance are 845, 1,055, and 485, respectively, that is, which needs additional corresponding reference to reject the previous relationship, and the fail-safe coefficients is more than “5k + 10” (Rhoades and Eisenberger, 2002). The corrected and uncorrected value *p* of Begg and Mazumdar rank correlation of each relationship is > 0.05, and the *p*-value of Egger’s test is also > 0.05, indicating that there is no serious publication bias.

### Heterogeneity Test

Heterogeneity test means the study of the effect size of each study to check whether there is heterogeneity. The study tested the heterogeneity of the relationship between workaholism and its dimensions and work performance. The results are shown in Table 3.

**TABLE 3:** Results of the heterogeneity test.

Variables relationship	*k*	*Q*	df	*p*	*I* ^2^	Tau-squared	*H*
WA → WP	26	750.857	25	0.000	96.670	0.077	30.034
WE → WP	21	1379.352	20	0.000	98.550	0.092	68.968
WC → WP	33	665.461	32	0.000	95.191	0.034	20.796

The results in Table 3 show that the value *p* is significant (*p* < 0.10) through the *Q* test of the effect size of each study, that is, the effect size in each study is heterogeneous. The value *I*^2^ ranged from 95.191 to 98.550, all > 75%, indicating that the effect size has a high heterogeneity (Higgins et al., 2003). The value of the Tau squared ranged from 0.034 to 0.092, indicating that 3.3–10.2% of the variation of the effect size can be used to calculate the weight. Value *H* is greater than 1.5, indicating that each effect size has high heterogeneity.

### Main Effect Test

Through the above theoretical analysis and heterogeneity test, we can find that the effect size of each study may be different. That is why the article selects the random effect model as the basic model for meta-analysis. The results of the main effect test of the article are shown in Table 4. Workaholism, working excessively, and working compulsively are significantly positively correlated with work performance (*r* = 0.140, *p*>0.05; *r* = 0.137, *p* < 0.05; *r* = 0.075, *p* < 0.05). The article assumes that 1, 2, and 3 are partially recognized.

**TABLE 4:** Random effect model analysis of the relationship between workaholism and work performance.

Variables relationship	k	N	Effect size and 95% confidence interval			Two-tailed test	
			Point estimation	Lower limit	Upper limit	value *Z*	value *P*
WA → WP	26	10,704	0.140	0.031	0.245	2.522	0.012
WE → WP	21	21,018	0.137	0.005	0.265	2.037	0.042
WC → WP	33	25,630	0.075	0.009	0.140	2.214	0.027

*K is the number of independent samples involved, and N is the number of research objects. The point estimation is the coefficient after the revised reliability.*

The article divides work performance into task performance and contextual performance according to the two-dimensional model, and comprehensively discusses the relationship between each dimension of workaholism and the two dimensions of work performance for further analysis. The random effect model is selected, and the effect analysis results are shown in Table 5. The relationship between the subdimension of workaholism and the subdimension of work performance is not consistent, mainly showing that working excessively and working compulsively failed to improve the task performance (*r* = 0.033, *p* > 0.05; *p* = 0.003, *p* > 0.05), but they were positively correlated with the contextual performance (*r* = 0.281, *p* < 0.001; *r* = 0.177, *p* < 0.001). The above results partially proved assumption 1 and failed assumption 2.

**TABLE 5:** Random effect model analysis of the relationship between working excessively and working compulsively, task performance and contextual performance.

Variables relationship	k	N	Effect size and 95% confidence interval			Two-tailed test	
			Point estimation	Lower limit	Upper limit	value *Z*	value *P*
WE → TP	13	10,097	0.033	−0.106	0.170	0.463	0.643
WE → CP	10	9,465	0.281	0.148	0.404	4.054	0.000
WC → TP	16	11,568	0.003	−0.082	0.088	0.070	0.944
WC → CP	17	11,527	0.177	0.114	0.238	5.492	0.000

*K is the number of independent samples involved, and N is the number of research objects. The point estimation is the coefficient after the revised reliability.*

### Subgroup Test

Through the heterogeneity test, we can find that there is a high heterogeneity among the effect sizes, where may exist moderator variables. The article regards the potential moderator variables as category variables in the process of data coding and extraction. The subgroup test is an effective method to explore the source of heterogeneity and handle the category moderator variables. Therefore, the article discusses the source of heterogeneity and the moderator effect of research characteristics through the subgroup test. The article mainly focuses on the moderator effects of the measurement tools of workaholism (DUWAS, WorkBAT, WART), cultural background (individualism, collectivism), and time-lag research (cross-sectional and longitudinal) on the relationship between various dimensions of workaholism and work performance (as shown in Tables 5–7).

**TABLE 6:** Moderator effect of the measurement tools of workaholism on the relationship between workaholism and work performance.

Variables relationship	Tools	*k*	*r*	95%CI	Q_w_	Q_b_	*P*
WE → WP	DUWAS	9	−0.068	[−0.228, 0.095]	701.258***	14.212	0.001
	WART	6	0.331	[0.201, 0.451]			
	WorkBAT	6	0.245	[−0.065, 0.512]			
WC → WP	DUWAS	9	0.001	[−0.127, 0.129]	506.568***	8.479	0.014
	WART	6	0.227	[0.119, 0.330]			
	WorkBAT	18	0.062	[−0.014, 0.138]			

Adoption of random effect model; Q_w_ indicates intergroup heterogeneity; Q_b_ indicates groups heterogeneity; ***p < 0.001. The same below.

**TABLE 7:** Moderator effect of cultural background on the relationship between workaholism and work performance.

Variables relationship	Cultural background	*k*	*R*	95%CI	Q_w_	Q_b_	*P*
WA → WP	Individualism	22	0.099	[−0.011, 0.207]	645.028***	2.488	0.115
	Collectivism	4	0.351	[0.053, 0.591]			
WE → WP	Individualism	15	0.136	[−0.051, 0.313]	1378.396***	0.001	0.971
	Collectivism	6	0.140	[−0.023, 0.296]			
WC → WP	Individualism	26	0.050	[−0.032, 0.131]	654.480***	2.353	0.125
	Collectivism	7	0.159	[0.046, 0.268]			

***p < 0.001.

The DUWAS scale is adopted for the relationship between the total score of workaholism and work performance, and the number of other scales does not exceed 3 so that there is no corresponding moderator test of the measurement tools. The results of Table 6 show that the measurement tools of workaholism can moderate the relationship between working excessively (Q_b_ = 14.212, *p* < 0.01) and working compulsively (Q_b_ = 8.479, *p* < 0.05) on work performance. There are significant differences in the above relationships even by three different scales. Furthermore, the relationship measured by WART is significantly different from that obtained by the other measurement tools. In other words, assumption 4 is true.

The results in Table 7 show that cultural background has no moderator role in the relationship between workaholism (Q_b_ = 2.488, *p* > 0.05), working excessively (Q_b_ = 0.001, *p* > 0.05) and working compulsively (Q_b_ = 0.001, *p* > 0.05) and work performance. In other words, there is no significant difference between collectivism and individualism. Assumption 5 is not favorable.

Since there are only 2 longitudinal studies involving the relationship between working excessively and work performance, less than 3 so there is no corresponding moderator effect test. The results in Table 8 show that the time-lag study did not significantly adjust the relationship between the total score of workaholism (Q_b_ = 1.742, *p* > 0.05) and working compulsively (Q_b_ = 1.742, *p* > 0.05) and work performance. In other words, there is no significant difference in the above relationship between the cross-sectional study and longitudinal study. Assumption 6 is not favorable.

**TABLE 8:** Moderator effect of the time-lag research on the relationship between workaholism and work performance.

Variables relationship	Time-lag research	*k*	*r*	95%CI	Q_w_	Q_b_	P
WA → WP	Cross-sectional	20	0.180	[0.060, 0.295]	707.555***	1.742	0.187
	Longitudinal	6	0.001	[−0.235, 0.237]			
WC → WP	Cross-sectional	28	0.070	[−0.007, 0.146]	665.309***	0.116	0.734
	Longitudinal	5	−0.009	[−0.031, 0.218]			

***p < 0.001.

## Discussion

### Relationship Between Workaholism and Work Performance

The relationship between workaholism and work-related results has always been the highlight of research. Since the proposal of the concept of workaholism, scholars have conducted numerous discussions on the relationship between workaholism and work performance. However, the research results are quite different (Ng et al., 2007; Serrano, 2015; Shimazu et al., 2015; Clark et al., 2016; Aziz et al., 2020; Balducci et al., 2020), which infringes on the comprehensive research on this topic. To date, there is no relevant research to clarify the cognition on this controversy. The article discusses the relationship between them for the first time with the help of meta-analysis technics. The results show that the total score of workaholism, working excessively, and working compulsively is positively correlated with work performance. The article gets an interesting conclusion after further analysis that working excessively and working compulsively cannot improve the task performance, but can significantly improve the contextual performance, which is consistent with the research conclusions in the past. The relevant conclusions obtained from the meta-analysis will be explained gradually.

First, there is a positive relationship between working excessively and work performance. Specifically, working excessively is not significantly correlated with task performance, but positively correlated with contextual performance, which proves the view of Gorgievski et al. (2010, 2014) and Van Beek et al. (2014). Workaholics devote time and numerous cognitive resources in their work, although some scholars suggested that the work efficiency of workaholics still needs to be improved. However, the long working hours of workaholics make up for the defect of low-work efficiency and can effectively improve work performance (better contextual performance). It can be found that the excellent performance of the workaholics is not their task performance, but contextual performance, which indirectly proves that the workaholics are concerned more with the views from others and the outside world as well as their high expectations for themselves. The typical opinion on workaholics is that their work scope exceeds the reasonable requirements of work or organization. The contextual performance is a direct way to judge whether they are excellent or not. Workaholics will do numerous extra behaviors to improve their relationship with others to get the recognition of others and the appreciation of their superiors, to get the praise of the outside world.

Second, working compulsively can also significantly improve work performance. Similar to working excessively, working compulsively is positively correlated with contextual performance, but not with task performance (Gorgievski et al., 2010; Lanaj et al., 2021). Working compulsively means the continuous and irresistible driven force of workaholics to work, which drives them to keep thinking all the time. Through the studies in the past, we can learn that working compulsively may be a harmful factor for the workaholics, which masks or offsets the positive effects of working excessively and the enjoyment of work (Gorgievski et al., 2010; Balducci et al., 2020). On the contrary, the results here show that it is not the case. Working compulsively can also improve work performance of the employees, especially in contextual performance. Working compulsively represents a perfectionist tendency of the workaholics, but the results show that strict requirement is not placed in task performance, but in contextual performance.

The article believes that the appearance of the above relationships may be related to the following reasons: first, the workaholics have a stronger sense of self-esteem, especially an organization-based sense of self-esteem, which also shows that workaholics care more about evaluation from others and organizations than their work tasks (Ng et al., 2007). They may not think about how to optimize their tasks, but how to improve the external evaluation no matter at work or during non-working hours. The behavior of caring more for the outside world will lead to efforts to improve the relationship with colleagues and leaders to improve work performance. Second, working compulsively reflects factors harmful to contextual performance (such as mental rigidity, reluctance to authorize, and negative emotions) (Robinson, 1999; Gorgievski et al., 2010; Balducci et al., 2020). However, it may also reflect the beneficial characteristics of contextual performance, including achievement orientation (Balducci et al., 2020) and stronger positive coping ability and emotional release ability (Shimazu and Schaufeli, 2009). Therefore, it may lead to more helping behaviors to others and higher contextual performance. Third, working compulsively is usually deemed as an introjected motivation. Compared with no motivation, the introjected motivation represented by work compulsion can also provide motivation to motivate employees to improve contextual performance, especially when the work is not attractive enough (Graves et al., 2012). Studies have shown that working compulsively also has positive results, including work satisfaction (Clark et al., 2016; Shkoler et al., 2017), organizational commitment (Clark et al., 2016) in addition to negative results. The positive emotion may cause the workaholics to care for colleagues, resulting in positive contextual performance. The article only gives alternative explanations for the emergence of the above relationship, which need further research to find the specific reasons.

Finally, workaholism also has a positive and significant relationship with work performance. Workaholics care more about resources at work due to being driven. The result of this high-intensity devotion is high work performance. As working excessively and working compulsively can generate higher work performance, the positive correlation between workaholism and work performance is understandable. But this result is inconsistent with the meta-analysis conclusion of Clark et al. (2016). We think these are mainly the following reasons. First, this research may have adopted a bigger sample. The meta-analysis sample includes 26 studies and 10,704 samples, while the meta-analysis of Clark et al. (2016) only includes 12 studies and 6,726 samples. Second, this inconsistency may be related to different types of performance. Just like this research results, workaholism has different effects on task performance and contextual performance. Clark et al. (2016) did not distinguish the dimensions of work performance, that is, they included different types of performance. However, our research excludes task performance and relational performance, and simply studies the relationship of workaholism on the work performance (only one dimension). Therefore, based on the above two reasons, the research results are not consistent with those of Clark et al. (2016).

### Moderating Effect

#### Measuring Tools of Workaholism

The results of the subgroup test showed that the measurement tool of workaholism significantly moderated the moderating variables of the relationship between working excessively and working compulsively and work performance. In other words, the relationship measured with the WART scale was significantly higher than that measured with the other scales, but there is no significant difference between the DUWAS scale and the WorkBAT scale.

The above situations may be caused by the following reasons. First, there is a large overlap between the DUWAS scale and the WorkBAT scale. Scholars Schaufeli et al. (2009b) directly cited WorkBAT and WART as the source of DUWAS scale topics when building the DUWAS scale, where the compulsive tendency dimension of WART (9 items) is deemed as the dimension of working excessively, while the driven dimension (8 items) is deemed as the dimension of working compulsively. Although the DUWAS scale with 17 questions was reduced to 10 items in the followed study, it did not adopt a similar essence of the DUWAS scale and WorkBAT scale. Therefore, more similarity on the topics may cause no significant difference between them, nor no possibility to significantly moderate the relationship between workaholism and work performance. Second, the WorkBAT scale and DUWAS scale are based on the same theoretical basis, that is, behavioral cognitive-affective theory. Other scales, including WART, WAQ, and BWAS, regarded work addiction as an addictive behavior viewed from the addiction theory. In the past, some scholars pointed out that work addiction and workaholism may not belong to the same concept (Griffiths et al., 2018), but most scholars regard them as the same. Due to the lack of research on the relationship between workaholism and work performance based on the WAQ and BWAS scales, it is necessary to use other scales to study more in the future.

#### Cultural Background

The results of the subgroup test showed that cultural background did not play a moderator role in the relationship between workaholism, working excessively, and working compulsively and work performance. In other words, there is no significant difference in the above relationship under the cultural background of collectivism or individualism.

The workaholics are forced more by their cognition, falling into the state of working excessively. The mechanism involving internal and external compulsive cognition leading to excessive behavior may not be influenced by external factors. The employees under collectivism are more willing to be responsible and work for the collective, but employees under individualism pay more attention to their growth and development (Hofstede et al., 2010). Therefore, there is no significant performance difference between them, although they may improve performance for different reasons. The same is true for workaholics. The relationship between workaholism and work performance will not be different due to cultural background on individualism or collectivism.

#### Time-Lag Research

The subgroup test results show that the time-lag study does not significantly moderate the relationship between workaholism, working compulsively, and work performance. In other words, there is no significant difference between the above relationship in the cross-sectional study and the longitudinal study. However, compared with the cross-sectional study, the above relationship in the longitudinal study is generally lower.

The article holds that the main reason for the above differences is that workaholism is a stable personal characteristic. Workaholism (working excessively and working compulsively) can be deemed as a continuous and stable work behavior and cognition. In other words, it will not be influenced by external time and will continuously and stably influence work performance. Ng et al. (2007) pointed out that the relationship between workaholism and work performance will be influenced by time. Seeing from the short-term, workaholics devote more working time than others, so their work performance will naturally be higher than that of the non-workaholics. However, seeing from the long term, excessive work devotion will damage the physical resources of the employees, leading to emotional exhaustion, generating worse physical and mental health, and social interpersonal relationships. In return, their work performance will be decreased. The meta-analysis results of the article do not fully prove this view. The relationship between workaholism and work performance may change with time, but this change is not significant. At the same time, there are relatively few longitudinal relationships between workaholism and work performance (only 6 and 3 items, respectively), which may deviate from the results. We shall explore the long-term effects of workaholism on the employees’ work performance and work behavior looking at the longitudinal view in the future.

### Research Limitations and Future Research Directions

The research limitations and prospects of the article mainly include the following: (1) The article does not involve the moderator role of gender, marital status, work position in the relationship between workaholism and work performance. The studies in the past have shown that men have higher workaholism than women, and married employees are also significantly higher than unmarried ones. Employees in management positions have higher workaholism than ordinary employees. We can conclude that gender, marital status, and work position may moderate the relationship between workaholism and work performance. (2) There are many kinds of measurement scales for workaholism. The article only selects WorkBAT, WART, and DUWAS scales, and does not involve the moderate effect of WAQ and BWAS. The main reason is that the number of relevant references adopting the WAQ and BWAS scales is limited to only 3, which may cause poor reliability of the moderator effect. The WAQ and BWAS scales can be used to explore the differences between workaholism and work performance to test whether there are differences in the features. (3) There are a few longitudinal research effect sizes in the subgroup analysis of the time-lag research, which may have a certain deviation from the results. Therefore, we can further investigate whether the subgroup analysis results are stable after the enrichment of relevant research in the future. (4) The work performance of the article only involves task performance and contextual performance, including organizational citizenship behavior, innovation performance, no involvement in the initiative behavior, deviant behavior, career prospect, and subjective career success. Therefore, the above variables can be included in future research to comprehensively explore the behavior results of workaholism.

## Conclusion

Through meta-analysis, the article finds that (1) workaholism, working excessively, and working compulsively are significantly positively correlated with work performance; (2) through a comprehensive analysis, the results show that working excessively and working compulsively are positively correlated with the contextual performance, but poor correlation with task performance; (3) the relationship between the total score of workaholism and its subdimensions and work performance is influenced by the measurement tools of workaholism, and no moderation by cultural background and time-lag research.

## Data Availability Statement

The original contributions presented in the study are included in the article/supplementary material, further inquiries can be directed to the corresponding author/s.

## Author Contributions

BC: conceptualization, methodology, software, validation, formal analysis, resources, data curation, writing—original draft, writing—review and editing, visualization, and project administration. JG: conceptualization, resources, data curation, writing—review and editing, project administration, fund acquisition. JG and BC completed the manuscript together. Both authors contributed to the article and approved the submitted version.

## Conflict of Interest

The authors declare that the research was conducted in the absence of any commercial or financial relationships that could be construed as a potential conflict of interest.

## Publisher’s Note

All claims expressed in this article are solely those of the authors and do not necessarily represent those of their affiliated organizations, or those of the publisher, the editors and the reviewers. Any product that may be evaluated in this article, or claim that may be made by its manufacturer, is not guaranteed or endorsed by the publisher.
